# Supplementary material for: Defective RNA polymerase III is negatively regulated by the SUMO-Ubiquitin-Cdc48 pathway
Source: eLife. 2018 Sep 7;7:e35447. doi: 10.7554/eLife.35447 (PMC6128692; doi:10.7554/eLife.35447)
Supplement: Figure 1—figure supplement 1—source data 1. [file elife-35447-fig1-figsupp1-data1.docx]

| **Gene** | **Protein** | **Allele name** | **Mutation** |
| --- | --- | --- | --- |
| *SMT3* | SUMO | *smt3-101* | K40E |
|  |  | *smt3-102* | F37L |
|  |  | *smt3-201* | D68H |
|  |  | *smt3-202* | R46M |
|  |  | *SMT3-Q56K** | Q56K |
| *AOS1* | SUMO E1 subunit | *aos1-101* | R21C |
| *UBA2* | SUMO E1 subunit | *uba2-101* | G147V |
| *UBC9* | SUMO E2 | *ubc9-201* | nt.G38C right before intron. |
|  |  |  | R13T, if spiced correctly. |
| *ULP1* | SUMO protease | *ulp1-101* | L338Stop |
|  |  | *ulp1-201* | Insertion at nt.66 |
| *ULP2* | SUMO protease | *ulp2-101* | C451F |
| *NUP84* | Nucleoporin | *nup84-101* | T-A at 39bp upstream of ORF |
|  | |  |  |
