# Supplementary material for: Defective RNA polymerase III is negatively regulated by the SUMO-Ubiquitin-Cdc48 pathway
Source: eLife. 2018 Sep 7;7:e35447. doi: 10.7554/eLife.35447 (PMC6128692; doi:10.7554/eLife.35447)
Supplement: Figure 2—figure supplement 1—source data 1. [file elife-35447-fig2-figsupp1-data1.docx]

| **Allele** | **Mutation** | **Location/Function** |
| --- | --- | --- |
| *rpc160-33* | T379I | Close to Rpc128 and catalytic site |
| *rpc160-58* | M809I | Close to Rpc128 |
| *rpc160-426* | E282K | Close to TFIIIB |
| *rpc160-419* | A880T | Bridge/NTP incorporation |
| *rpc160-480* | G1098D | Trigger loop/NTP incorporation |
| *rpc160-628* | R365K | Close to DNA |
| *rpc160-211* | G606S | Rpb8 |
| *rpc160-85* | G1297D | Close to DNA |
| *rpc128-202* | A704T | Close to RNA |
| *rpc128-578* | D501N | Close to RNA |
