# Supplementary material for: Defective RNA polymerase III is negatively regulated by the SUMO-Ubiquitin-Cdc48 pathway
Source: eLife. 2018 Sep 7;7:e35447. doi: 10.7554/eLife.35447 (PMC6128692; doi:10.7554/eLife.35447)
Supplement: Figure 2—figure supplement 3—source data 1. [file elife-35447-fig2-figsupp3-data1.docx]

|  | **Mutation in** | **Mutation in** | **5FOA 30°C** | | **5FOA 37°C** | |
| --- | --- | --- | --- | --- | --- | --- |
| **Gene** | **human protein** | **yeast protein** | ***SIZ1+*** | ***siz1Δ*** | ***SIZ1+*** | ***siz1 Δ*** |
| *RPC160* | D372N | D384N | + | + | + | + |
| (*POLR3A*) | A387G | A399G | + | + | + | + |
|  | T553I | S565I | + | + | + | + |
|  | Q599K | Q608K | -/+ | +/- | -/+ | +/- |
|  | E644K | Q658K | + | + | + | + |
|  | G672E | G686E | + | + | + | + |
|  | C724Y | C738Y | + | + | + | + |
|  | N775I | N789I | + | + | + | + |
|  | G784S | G798S | + | + | + | + |
|  | M852V | I866V | + | + | + | + |
|  | R1005C | R1061C | + | + | + | + |
|  | R1005H | R1061H | + | + | + | + |
|  | G1240S | G1308S | + | + | + | + |
|  | E1261K | E1329K | - | - | - | - |
|  | A1331T | A1399T | + | + | + | + |
|  | D372N, N775I | D384N, N789I | - | +/- | - | -/+ |
|  | Q599K, G1240S | Q608K, G1308S | -/+ | +/- | - | - |
| *RPC128* | T503K | T521K | + | + | + | + |
| (*POLR3B*) | V523E | I541E | + | + | + | + |
|  | D895N | D910N | + | + | + | + |
|  | L1012P | L1027P | -/+ | + | - | +/- |
|  | L1117V | L1132V | + | + | + | + |
|  | V523E, D895N | I541E, D910N | - | - | - | - |
|  | V523E, L1012P | I541E, L1027P | - | - | - | - |
|  | V523E, L1117V | I541E, L1132V | - | - | - | - |
| *BRF1* | R223W | R218W | - | - | - | - |
| (*BRF1*) | S226L | A221L | + | + | + | + |
|  | T259M | T254M | - | +/- | - | -/+ |
|  | P292H | P288H | +/- | + | -/+ | + |
