# Supplementary material for: Defective RNA polymerase III is negatively regulated by the SUMO-Ubiquitin-Cdc48 pathway
Source: eLife. 2018 Sep 7;7:e35447. doi: 10.7554/eLife.35447 (PMC6128692; doi:10.7554/eLife.35447)
Supplement: Figure 4—figure supplement 1—source data 1. [file elife-35447-fig4-figsupp1-data1.docx]

| Hendriks IA, et al. | | Tammsalu T, et al. |  | Lamoliatte F, et al. | |
| --- | --- | --- | --- | --- | --- |
| Modified protein | # of sites | Modified protein | # of sites | Modified protein | # of sites |
| RPA34 (Pol I) | 2 | RPA34 (Pol I) | 1 | RPC4 (Pol III) | 1 |
| RPB1 (Pol II) | 4 | RPC4 (Pol III) | 7 |  |  |
| RPB3 (Pol II) | 1 | RPC6 (Pol III) | 1 |  |  |
| RPC3 (Pol III) | 1 | RPABC3 (Pol I, II, III) | 1 |  |  |
| RPC4 (Pol III) | 7 |  |  |  |  |
| RPC5 (Pol III) | 2 |  |  |  |  |
| RPC6 (Pol III) | 1 |  |  |  |  |
| RPAC1 (Pol I, III) | 1 |  |  |  |  |
