# Supplementary material for: Defective RNA polymerase III is negatively regulated by the SUMO-Ubiquitin-Cdc48 pathway
Source: eLife. 2018 Sep 7;7:e35447. doi: 10.7554/eLife.35447 (PMC6128692; doi:10.7554/eLife.35447)
Supplement: Supplementary file 1. [file elife-35447-supp1.docx]

| **Strain Name** | **Genotype** |
| --- | --- |
| ZBY116 | *MATa his4-912δ lys2-128δ suc2Δuas(-1900/-390) ura3-52 leu2Δ1 trp1Δ63 mot1-301* |
| ZOY261 | *MATa ade2 ade3 can1Δ::FUR1::natMX4 ura3 leu2 trp1Δ63 his3Δ1 lys2-128δ* |
| ZBY694 | *MATa his4-912δ lys2-128δ suc2Δuas(-1900/-390) ura3-52 leu2Δ1 trp1Δ63 rpc128Δ2::natMX4 [pF92 (CEN URA3 RPC128)] [pZW393 (CEN LEU2 rpc128-A704T)]* |
| ZBY282 | *MATα suc2Δuas(-1900/-390) ura3-52 leu2Δ1 trp1Δ63 rpc160-M809I-3HA-kanMX6 [pZW328 (CEN URA3 RPC160)] his4-912δ* |
| ZBY306 | *MATa suc2Δuas(-1900/-390) ura3-52 leu2Δ1 trp1Δ63 his4-912δ lys2-128δ siz1Δ::TRP1* |
| ZBY301 | *MATa his4-912d lys2-128d suc2Δuas(-1900/-390) ura3-52 trp1Δ63 siz2Δ::KAN* |
| ZBY259 | *MATα his4-912δ suc2Δuas(-1900/-390) ura3-52 leu2Δ1 trp1Δ63 rpc160Δ1::TRP1 [pZW328 (CEN URA3 TRS33 RPC160)]* |
| ZBY419 | *MATa suc2Δuas(-1900/-390) ura3-52 leu2Δ1 trp1Δ63 rpc160Δ1::TRP1 [pZW328 (CEN URA3 RPC160)] siz1Δ::TRP1 his4-912δ* |
| ZBY788 | *MATα suc2Δuas(-1900/-390) ura3-52 leu2Δ1 trp1Δ63 rpc31Δ1::kanMX6 [pZW420 (CEN URA3 RPC31)] lys2-128δ* |
| ZBY829 | *MATα suc2Δuas(-1900/-390) ura3-52 leu2Δ1 trp1Δ63 rpc31Δ1::kanMX6 [pZW420 (CEN URA3 RPC31)] siz1Δ::TRP1 lys2-128δ* |
| ZBY780 | *MATα suc2Δuas(-1900/-390) ura3-52 leu2Δ1 trp1Δ63 rpb1Δ1::kanMX6 [pZW413 (CEN URA3 RPB1)]* |
| ZBY823 | *MATα suc2Δuas(-1900/-390) ura3-52 leu2Δ1 trp1Δ63 rpb1Δ1::kanMX6 [pZW413 (CEN URA3 RPB1)] siz1Δ::TRP1* |
| ZBY1065 | *MATa suc2Δuas(-1900/-390) his4-912d lys2-128d ura3-52 trp1Δ63 rpa190Δ1::kanMX6 [pZW544 (CEN URA3 RPA190)] leu2Δ1* |
| ZBY1067 | *MATa suc2Δuas(-1900/-390) his4-912d lys2-128d ura3-52 trp1Δ63 rpa190Δ1::kanMX6 [pZW544 (CEN URA3 RPA190)] siz1Δ::TRP1 leu2Δ1* |
| ZBY739 | *MATα suc2Δuas(-1900/-390) ura3-52 leu2Δ1 trp1Δ63 kns1Δ1::kanMX6 his4-912d* |
| ZBY747 | *MATα suc2Δuas(-1900/-390) ura3-52 leu2Δ1 trp1Δ63 mck1Δ ::natMX4 his4-912d* |
| ZBY313 | *MATa suc2Δuas(-1900/-390) ura3-52 leu2Δ1 trp1Δ63 maf1Δ1::natMX4 lys2-128d* |
| ZBY18 | *MATa his4-912δ lys2-128δ suc2Δuas(-1900/-390) ura3-52 leu2Δ1* |
| ZBY445 | *MATa rpc160-M809I-3HA::kanMX6 ura3-52 lys2-128δ suc2Δuas(-1900/-390) trp1Δ63* |
| ZBY346 | *MATa his4-912δ suc2Δuas(-1900/-390) ura3-52 leu2Δ1 trp1Δ63 rpc160-M809I-3HA-kanMX6 siz1Δ::TRP1* |
| ZBY370 | *MATa suc2Δuas(-1900/-390) ura3-52 leu2Δ1 trp1Δ63 his4-912d maf1Δ1::natMX4 rpc160-M809I-3HA-kanMX6* |
| ZBY439 | *MATa suc2Δuas(-1900/-390) ura3-52 leu2Δ1 trp1Δ63 his4-912d maf1Δ1::natMX4 rpc160-M809I-3HA-kanMX6 siz1Δ::TRP1* |
| ZBY434 | *MATa suc2Δuas(-1900/-390) ura3-52 leu2Δ1 trp1Δ63 his4-912d maf1Δ1::natMX4 siz1Δ::TRP1* |
| ZOY341 | *MATα ura3 leu2 trp1Δ63 rpc160-M809I-Flag::TRP1 smt3Δ::kan [pZW357 (2μ LEU2 smt3-I96R)] slx5Δ::URA3 his4-912δ* |
| ZOY504 | *MATα ura3 leu2 trp1Δ63 his3Δ1 smt3Δ::TRP1 [pZW508 (2μ LEU2 GFP-SMT3)] slx5Δ::URA3 met15Δ0 suc2Δuas(-1900/-390) lys2-128δ* |
| ZOY505 | *MATα ura3 leu2 trp1Δ63 met15Δ0 his3Δ1 rpc160-M809I-Flag::TRP1 slx5Δ::URA3 smt3Δ::kan [pZW508 (2μ LEU2 GFP-SMT3)]* |
| ZBY1106 | *MATa suc2Δuas(-1900/-390) ura3-52 leu2Δ1 trp1Δ63 lys2-128δ rpc53Δ2::hphMX4 [pZW551 (CEN URA3 RPC53)]* |
| ZOY465 | *MATa ura3 leu2 trp1Δ63 lys2-128δ rpc128-A704T [pF92 (CEN URA3 RPC128)]* |
| ZBY1056 | *MATa suc2Δuas(-1900/-390) ura3-52 leu2Δ1 trp1Δ63 RPC53-Flag::natMX4 his4-912d* |
| ZBY1054 | *MATα suc2Δuas(-1900/-390) ura3-52 leu2Δ1 trp1Δ63 RPC53-Flag::natMX4 rpc160-M809I-3HA::kanMX6* |
| ZBY1257 | *MATα suc2Δuas(-1900/-390) ura3-52 leu2Δ1 trp1Δ63 RPC53-Flag::natMX4 siz1Δ::TRP1 his4-912d* |
| ZBY1479 | *MATa suc2Δuas(-1900/-390) ura3-52 leu2Δ1 trp1Δ63 RPC53-Flag::natMX4 siz2Δ::kan lys2-128d* |
| ZBY1263 | *MATa suc2Δuas(-1900/-390) ura3-52 leu2Δ1 trp1Δ63 RPC53-Flag::natMX4 ulp2-101 rpc160-M809I-3HA::kanMX6 his4-912d* |
| ZBY660 | *MATa suc2Δuas(-1900/-390) ura3-52 leu2Δ1 trp1Δ63 rpc128Δ2::natMX4 [pF92 (CEN URA3 RPC128)] siz1Δ::TRP1 his4-912δ lys2-128δ* |
| ZBY661 | *MATa suc2Δuas(-1900/-390) ura3-52 leu2Δ1 trp1Δ63 rpc128Δ2::natMX4 [pF92 (CEN URA3 RPC128)] his4-912δ lys2-128δ* |
| ZBY1233 | *MATa suc2Δuas(-1900/-390) ura3-52 leu2Δ1 trp1Δ63 brf1Δ1::kanMX6 [pZW679 (CEN URA3 BRF1)] siz1Δ::TRP1* |
| ZBY1235 | *MATa suc2Δuas(-1900/-390) ura3-52 leu2Δ1 trp1Δ63 brf1Δ1::kanMX6 [pZW679 (CEN URA3 BRF1)]* |
| ZBY91 | *MATa his4-912d lys2-128d suc2Δuas(-1900/-390) ura3-52 leu2Δ1 trp1Δ63 slx5Δ::URA3* |
| ZBY92 | *MATa his4-912d lys2-128d suc2Δuas(-1900/-390) ura3-52 leu2Δ1 trp1Δ63 slx8Δ::TRP1* |
| ZBY290 | *MATα suc2Δuas(-1900/-390) ura3-52 leu2Δ1 trp1Δ63 rpc160-G1297D-3HA-kanMX6 [pZW328 (CEN URA3 RPC160)] his4-912d* |
| ZOY197 | *MATa his3Δ1 ura3Δ0 leu2Δ0 met15Δ0 ubc4Δ::KAN* |
| PJ69-4A | *MATa his3Δ200 leu2-3,112 trp1-901 ura3-52 gal4Δ gal80Δ LYS2::GAL1-HIS3 ade2::GAL2-ADE2 met2::GAL7-LacZ* |
| ZBY364 | *MATa suc2Δuas(-1900/-390) ura3-52 leu2Δ1 trp1Δ63 rpc160-G1297D-3HA-kanMX6 slx5Δ::URA3 his4-912d* |
| ZBY1145 | *MATα suc2Δuas(-1900/-390) ura3-52 leu2Δ1 trp1Δ63 cdc48Δ2::hphMX4 [pZW880 (CEN LEU2 cdc48-3)] his4-912d* |
| ZBY1451 | *MATα suc2Δuas(-1900/-390) ura3-52 leu2Δ1 trp1Δ63 cdc48Δ2::hphMX4 [pZW881 (CEN LEU2 cdc48-sim)] his4-912d* |
| ZBY1460 | *MATa suc2Δuas(-1900/-390) ura3-52 leu2Δ1 trp1Δ63 ufd1Δ2::hphMX4 [pZW879 (CEN LEU2 ufd1-sim)] lys2-128d* |
| ZBY1513 | *MATa suc2Δuas(-1900/-390) ura3-52 leu2Δ1 trp1Δ63 rpc160-M809I-3KR-3HA::kanMX6 his4-912d* |
| ZBY1545 | *MATa suc2Δuas(-1900/-390) ura3-52 leu2Δ1 trp1Δ63 rpc160-G1297D-3KR-3HA::kanMX6 his4-912d* |
| ZBY1537 | *MATa suc2Δuas(-1900/-390) ura3-52 leu2Δ1 trp1Δ63 rpc160-3KR-3HA::kanMX6 his4-912d* |
| ZBY1118 | *MATα suc2Δuas(-1900/-390) ura3-52 leu2Δ1 trp1Δ63 lys2-128d rpc53Δ2::hphMX4 [pZW577 (CEN LEU2 rpc53-3KR-Flag)]* |
| ZBY591 | *MATa suc2Δuas(-1900/-390) ura3-52 leu2Δ1 trp1Δ63 pdr5Δ1::natMX4 his4-912d* |
| ZBY521 | *MATa suc2Δuas(-1900/-390) ura3-52 leu2Δ1 trp1Δ63 rpc160-M809I-Flag::TRP1 slx5Δ::URA3* |
| ZBY1466 | *MATa suc2Δuas(-1900/-390) ura3-52 leu2Δ1 trp1Δ63 cdc48Δ2::hphMX4 [pZW898 (CEN LEU2 cdc48-3-Flag)] lys2-128d* |
| ZBY1496 | *MATα suc2Δuas(-1900/-390) ura3-52 leu2Δ1 trp1Δ63 cdc48Δ2::hphMX4 [pZW898 (CEN LEU2 cdc48-3-Flag)] rpc160-M809I-3HA::kanMX6 his4-912d lys2-128d* |
| ZBY1574 | *MATa his4-912d suc2Δuas(-1900/-390) ura3-52 leu2Δ1 trp1Δ63 rpc160-G1297D-3HA-kanMX6 slx8Δ::TRP1 lys2-128d [pZW328 (CEN URA3 RPC160)]* |
