# Supplementary material for: Defective RNA polymerase III is negatively regulated by the SUMO-Ubiquitin-Cdc48 pathway
Source: eLife. 2018 Sep 7;7:e35447. doi: 10.7554/eLife.35447 (PMC6128692; doi:10.7554/eLife.35447)
Supplement: Supplementary file 2. [file elife-35447-supp2.docx]

| **Plasmid name** | **Markers** |
| --- | --- |
| pRS415 | *Amp CEN LEU2* |
| pRS425 | *Amp 2μ LEU2* |
| pF5 | *Amp CEN LEU2 SMT3* |
| pZW81 | *Amp CEN LEU2 MOT1* |
| pZW311 | *Amp CEN LEU2 SMT3-Q56K* |
| pZW321 | *Amp CEN URA3 ADE3 SMT3-Q56K* |
| pAK21-1 | *Amp CEN TRP1 ade3-pink* |
| pZW548 | *Amp CEN LEU2 RPA190* |
| pZW890 | *Amp CEN LEU2 rpa190-G1194D* |
| pZW411 | *Amp CEN LEU2 RPB1* |
| pZW8910 | *Amp CEN LEU2 rpb1-G1073D* |
| pZW326 | *Amp CEN LEU2 RPC160* |
| pZW371 | *Amp CEN LEU2 rpc160-G1098D* |
| pZW400 | *Amp CEN LEU2 rpc160-112* |
| pZW401 | *Amp CEN LEU2 rpc160-270* |
| pZW873 | *Amp CEN LEU2 PGAL1-RPC160-Flag* |
| pZW419 | *Amp CEN LEU2 RPC31* |
| pZW423 | *Amp CEN LEU2 rpc31-236* |
| pZW725 | *Amp CEN LEU2 RPC53* |
| pZW564 | *Amp CEN LEU2 RPC53-Flag* |
| pZW565 | *Amp CEN LEU2 rpc53-K51R-Flag* |
| pZW566 | *Amp CEN LEU2 rpc53-K115R-Flag* |
| pZW567 | *Amp CEN LEU2 rpc53-K216R-Flag* |
| pZW569 | *Amp CEN LEU2 rpc53-K236R-Flag* |
| pZW570 | *Amp CEN LEU2 rpc53-K115,236R-Flag* |
| pZW575 | *Amp CEN LEU2 rpc53-K51,115R-Flag* |
| pZW576 | *Amp CEN LEU2 rpc53-K51,236R-Flag* |
| pZW577 | *Amp CEN LEU2 rpc53-K51,115,236R-Flag* |
| pZW568 | *Amp CEN LEU2 rpc53-K322,325R-Flag* |
| pZW571 | *Amp CEN LEU2 rpc53Δ2-275-Flag* |
| pZW384 | *Amp CEN LEU2 RPC128* |
| pZW596 | *Amp CEN LEU2 smt3ΔGG-rpc53-K51,115,236R-Flag* |
| pZW630 | *Amp CEN LEU2 rpc160-S565I* |
| pZW579 | *Amp CEN LEU2 rpc160-D384N, N789I* |
| pZW640 | *Amp CEN LEU2 rpc160-E1329K* |
| pZW387 | *Amp CEN LEU2 rpc128-I541E* |
| pZW624 | *Amp CEN LEU2 rpc128-L1027P* |
| pZW678 | *Amp CEN LEU2 BRF1* |
| pZW680 | *Amp CEN LEU2 brf1-R218W* |
| pZW681 | *Amp CEN LEU2 brf1-A221L* |
| pZW682 | *Amp CEN LEU2 brf1-T254M* |
| pZW683 | *Amp CEN LEU2 brf1-P288H* |
| pZW144 | *Amp 2μ LEU2 SMT3* |
| pGADT7 | *Amp 2μ LEU2GAL4-AD* |
| pGBKT7 | *Kan 2μ TRP1 GAL4-BD* |
| pCS6514 | *Amp 2μ LEU2 GAL4-AD-SLX5* |
| pZW584 | *Kan 2μ TRP1 GAL4-BD-RPC53* |
| pZW591 | *Kan 2μ TRP1 GAL4-BD-rpc53-3KR* |
| pZW592 | *Kan 2μ TRP1 GAL4-BD-rpc53ΔN (Δ2-275)* |
| pZW331 | *Amp CEN URA3 RPC160-HA* |
| pZW332 | *Amp CEN URA3 rpc160-M809I-HA* |
| pZW968 | *Amp CEN URA3 rpc160-M809I-3KR-HA* |
| pZW938 | *Amp CEN LEU2 SIZ1-Myc* |
| pZW940 | *Amp CEN LEU2 PCUP1-siz1ΔSAP-Myc (Δ34-68)* |
| pZW941 | *Amp CEN LEU2 PCUP1-siz1-SAP*-Myc* |
| pGP776 | *Amp CEN LEU2 SIZ1* |
| pZW903 | *Amp CEN LEU2 SIZ1-Flag* |
| pZW904 | *Amp CEN LEU2 PCUP1-siz1ΔSAP-Flag* |
| pZW942 | *Amp CEN LEU2 PCUP1-siz1-SAP*-Flag* |
| pZW986 | *Amp 2μ LEU2 HA-SLX8* |
| pZW993 | *Amp 2μ LEU2 PCUP1-HA-slx8ΔN (Δ2-163)* |
| pZW900 | *Amp CEN LEU2 PCUP1-RPC160-HA* |
| pZW901 | *Amp CEN LEU2 PCUP1-rpc160-M809I-HA* |
