# Supplementary material for: Defective RNA polymerase III is negatively regulated by the SUMO-Ubiquitin-Cdc48 pathway
Source: eLife. 2018 Sep 7;7:e35447. doi: 10.7554/eLife.35447 (PMC6128692; doi:10.7554/eLife.35447)
Supplement: Supplementary file 3. — The same primers were used in RNA level measurement and in chromatin IP experiments. * Used as gene-specific primer in reverse transcription. [file elife-35447-supp3.docx]

| **Gene** | **Sequence** |
| --- | --- |
| tM(CAU) | Forward: GCTTCAGTAGCTCAGTAGGAA |
|  | Reverse: TGCTCCAGGGGAGGTTC* |
| tG(UCC) | Forward: GGGCGGTTAGTGTAGTGGTT |
|  | Reverse: TGAGCGGTACGAGAATCGAA* |
| tL(CAA) | Forward: GGTTGTTTGGCCGAGCG |
|  | Reverse: TGGTTGCTAAGAGATTCGAACTC* |
| pre-tL(CAA)A | Forward: GGTTGTTTGGCCGAGCG |
|  | Reverse: CCCACAGTTCACTGCGGTC |
| ACT1 | Forward: CTGGTATGTTCTAGCGCTTG |
|  | Reverse: ATCTCTCGAGCAATTGGGAC |
| TDH3 | Forward: CTGGTGAAGTTTCCCACGAT |
| (GAPDH) | Reverse: TCGTTAACACCCATGACGAA |
| 18S rDNA | Forward: TCGACCCTTTGGAAGAGATG |
|  | Reverse: CTCCGGAATCGAACCCTTAT |
|  | |
